# Use of RT-Defective HIV Virions: New Tool to Evaluate Specific Response in Chronic Asymptomatic HIV-Infected Individuals

**DOI:** 10.1371/journal.pone.0058927

**Published:** 2013-03-14

**Authors:** Alberto Crespo Guardo, Carmen Álvarez-Fernández, Hodei Arberas, Javier García-Pérez, Felipe García, Manuel Enric Bargalló, María José Maleno, José María Gatell, Beatriz Mothe, José Alcami, Sonsoles Sánchez-Palomino, Montserrat Plana

**Affiliations:** 1 Retrovirology and Viral Immunopathology Laboratory, Institut d´Investigacions Biomèdiques August Pi I Sunyer (IDIBAPS), Hospital Clínic, University of Barcelona, Barcelona, Spain; 2 Catalan Program for HIV Vaccine Development (HIVACAT), Barcelona, Spain; 3 Infectious Diseases Unit, Hospital Clínic, IDIBAPS, University of Barcelona, Barcelona, Spain; 4 AIDS Immunopathology Unit. National Center of Microbiology, Instituto de Salud Carlos III, Madrid, Spain; 5 Institut de Recerca de la SIDA IrsiCaixa – HIVACAT, Hospital Germans Trias i Pujol, Badalona, Barcelona, Spain; Blood Systems Research Institute, United States of America

## Abstract

**Background:**

Generation of new reagents that can be used to screen or monitor HIV-1-specific responses constituted an interesting field in the development of HIV vaccines to improve their efficacy.

**Methods:**

We have evaluated the specific T cell response against different types of NL4-3 virions (including NL4-3 aldrithiol-2 treated, NL4-3/ΔRT and R5 envelopes: NL4-3/ΔRT/ΔEnv[AC10] and NL4-3/ΔRT/ΔEnv[Bal]) and against pools of overlapping peptides (15 mer) encompassing the HIV-1 Gag and Nef regions. Cryopreserved PBMC from a subset of 69 chronic asymptomatic HIV positive individuals have been employed using different techniques including IFN-γ ELISPOT assay, surface activation markers and intracellular cytokine staining (ICS) by flow cytometry.

**Results:**

The differential response obtained against NL4-3 aldrithiol-2 treated and NL4-3/ΔRT virions (25% vs 55%, respectively) allow us to divide the population in three groups: “*full-responders*” (positive response against both viral particles), *“partial-responders”* (positive response only against NL4-3/ΔRT virions) and “*non-responders*” (negative responses). There was no difference between X4 and R5 envelopes. The magnitude of the total responses was higher against NL4-3/ΔRT and was positively correlated with gender and inverse correlated with viral load. On the contrary CD4+ T cell count was not associated with this response. In any case responses to the viruses tended to be lower in magnitude than those detected by the overlapping peptides tested. Finally we have found an increased frequency of HLA-B27 allele (23% vs 9%) and a significant reduction in some activation markers (CD69 and CD38) on T cells surface in responders vs non-responders individuals.

**Conclusions:**

In summary these virions could be considered as alternative and useful reagents for screening HIV-1-specific T cell responses in HIV exposed uninfected people, HIV infected patients and to assess immunogenicity of new prototypes both *in vitro* and in vaccine trials, by a feasible, simply, effective and low cost assay.

## Introduction

During natural course of HIV-1 infection there is a gradual increase in HIV-1 RNA viremia in parallel with impairment on functional HIV-1-specific CD4+ and CD8+ T cell responses. At the end of this process a state of severe immunodeficiency emerges in HIV-1 infected individuals. Recent studies suggest that the induction of HIV-1-specific CD4+ T helper cells and polyfunctional CD8+ T cells could play an important role in controlling HIV-1 replication and spread in patients with HIV-1 infection [1]–[4]. Thus, a vaccine regimen capable of inducing broadly reactive HIV-specific CD4+ and CD8+ T-cell responses may be required.

One of the major challenges in vaccine development is the limited understanding of the correlates of immune protection [5]. So, an accurate and efficient monitoring and characterisation of HIV-1-specific T-cells is very important to understand the pathogenesis of HIV-1 infection as well as to determine potential efficacy of HIV-1 vaccines and immune-based therapies. Nowadays, technologies used to quantify and analyze HIV-specific T cell responses are quite efficient but expensive and laborious [6]–[7]. In fact, and due to the high variability of the HIV, most of these techniques do not allow detecting immune T cell responses against different variable sequences of the autologous virus. So, there is an important need to develop new reagents able to be more closely related to the HIV native virus and to simulate or use a mechanism of infection more similar to the physiological one.

In this sense and attending to this differential and important role for RT, our group has recently reported the construction and characterization of retrotranscriptase defective virions (NL4-3/ΔRT) [8] to improve safety as a future candidate for being used in HIV therapeutic vaccines. These attenuated constructs prevent viral integration and further replication thus avoiding evolution towards a pathogenic variant with a total deletion of the gene coding RT. On the other side, these defective virions preserved the structural integrity and are able to interact with antigen presenting cells, thus allowing protein processing and T cell presentation. The primary objective of the present work was to further analyse the *in vitro* immunogenicity of these virions throwing light on some aspects such as the importance of different tropism (X4 and R5 variants) or the magnitude of their response in comparison to the elicited by a pool of peptides encompassing Gag and Nef proteins. Herein we showed that NL4-3/ΔRT viral particle result an efficient inductor of the cellular immune response against HIV-1 when used as a reagent *in vitro* in PBMC cultures. Additionally, we also evaluated some clinical and immunological features of HIV patients able to respond positively to this attenuated virus. According to their safety profile and their relative low cost in comparison to other compounds they could be considered as a future candidate to be used as an effective immunogen in therapeutic approaches or as an additional and useful reagent for screening HIV-1-specific T cell responses in HIV infected patients and to assess vaccine immunogenicity both *in vitro* and *in vivo*.

## Results

### Retrotranscriptase Defective Virions Elicit Strong Specific T cell Immune Response against HIV-1

We have developed a classical IFN-γ-ELISPOT assay to measure the immunogenic capacity *in vitro* of viral particles produced from different NL4.3-based constructs. We have compared both the proportion of positive responses elicited and the intensity of the immune responses induced by a wild type NL4-3 virus AT-2 inactivated (NL4-3+AT-2, o referred as wild type) with RT-deleted genomes carrying both X4 (NL4-3/ΔRT) and R5 envelope genes (NL4-3/ΔRT/ΔEnv[AC10]; NL4-3/ΔRT/ΔEnv[Bal]) to assess the potential importance of co-receptor use and RT deletion in triggering cellular responses.

By contrast, we did not include in our assay a non-attenuated NL4-3 virus to avoid the potentially confounding effects of a productive infectious virus able to replicate and integrate.

**Figure 1:**
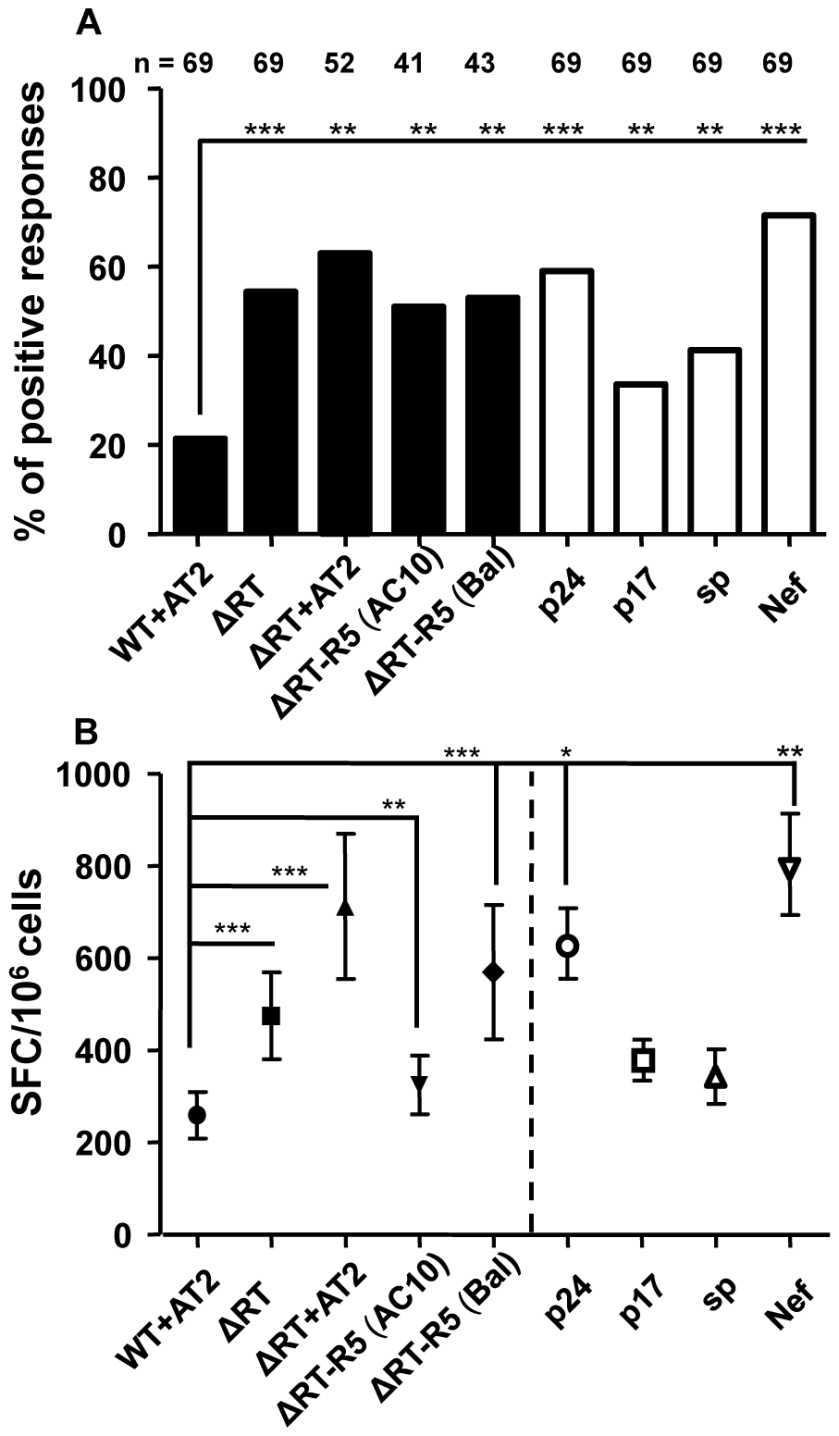
Comparison of frequencies and magnitudes of responses against the different viral particles and peptides pools tested. (**A**) Bars show the percentage of individuals with positive response to the different constructs (Black bars) and the gag p24, p17 and small proteins (sp) and nef peptide pools (White bars) among the samples of HIV+ individuals tested. (**B**) Each symbol represents the mean of SFC (spot forming cells/10^6^ cells) ± SEM counted per condition after background subtraction. *P* values were calculated using the *Wilcoxon Matched*-Pairs Ranks test for continuous variables and χ2-test for categorical variables. The magnitude of response was significantly different among indicated stimuli (*p<0.05; **p<0.01; ***p<0.001).

As it is shown in figure 1A the proportion of patients with positive immune responses against NL4-3/ΔRT (ΔRT) virions (55%) was significantly higher than to wild type (25%) (p<0.001) [8] independently of the use of R5 or X4 receptors by viral envelopes (51–53%). The magnitude of the response (SFC/10^6^ cells) elicited by both the X4 and R5 ΔRT variants was also higher than with wild type virus (p<0.001 and p<0.01, respectively), fact that confirmed that this difference was also applicable to the intensity of the response (fig. 1B).

We also assessed the T cell response to a ΔRT virus treated with AT-2 to look at the potential effect of this pretreatment on immune responses. As shown in Fig. 1A and B, no deleterious effect was observed when we analyzed both the frequency and the magnitude of positive cellular responses obtained when compared to ΔRT virus non previously pretreated. Of note, we even observed a better and higher response when our defective ΔRT construct was additionally chemically inactivated.

Lastly, we also evaluated in parallel the response to Gag p24, Gag p17, Gag small proteins (sp) and Nef peptide pools. As is shown in Fig. 1A, percentages of HIV-infected individuals who responded to theses peptide pools were significantly greater than to the wild type whereas they were similar to the rate of response to the different constructs of our defective virions (ΔRT). Moreover, concerning the magnitude, response to Gag p24 and Nef peptide pools was higher than to both wild type and ΔRT (Fig. 1B).

### ICS Assay Confirmed ELISPOT Findings and Detected Positive Responses in both Lineages: CD4 and CD8

**Figure 2:**
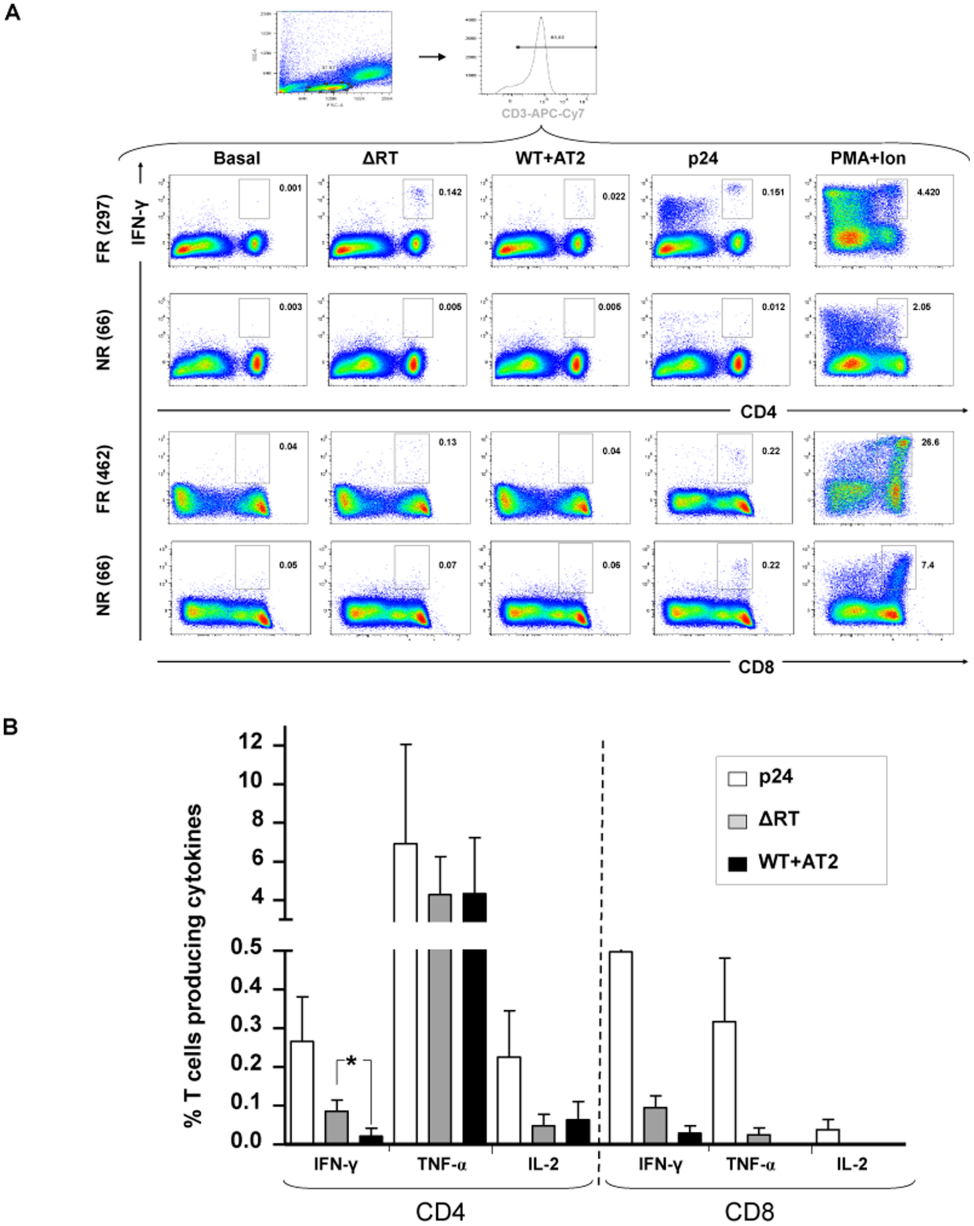
Example of IFN-γ response in CD4 and CD8 T cell subsets from different individuals: full responder (FR) vs non-responder (NR). (**A**) Representative dot plots of gating strategy used and cytokine responses detected using different stimuli. Box indicated the percentage of IFN-γ+ T cells. PMA-Ionophore was used as a positive control. (**B**) Evaluation of cytokine production in both subsets (CD4 and CD8). Percentage of T cells producing IFN-γ, TNF-α or IL-2 in response to virions (ΔRT or WT+AT2) or to a pool of peptides encompassing p24 is depicted.

HIV-1 specific immune responses were also measured by a 6 colour ICS assay in a subgroup of 21 HIV-infected patients previously tested by ELISPOT (Fig. 2A). Total responses against NL4-3/ΔRT were confirmed on 12 out of 16 (75%) of the ELISPOT positive responders to these particles whereas only 4 out of 13 (31%) wild type responders to ELISPOT were detected by ICS. These results indirectly confirmed the higher and earlier response obtained against ΔRT immature viral particles in comparison with wild type AT-2 inactivated virus. In fact negative response were described on 5 (100%) of the 5 negative ELISPOT responders illustrating the specificity and reproducibility of the detected response. On the other side, and as control, cytokine production by HIV-p24-stimulated T cells was also measured in parallel, and the results showed positive response in 15 out of 21 (71%) patients tested.

We next wanted to asses the different T cell lineage able to respond to our ΔRT virus, and found that CD4+ T-cell responses were present in 44% of the patients after stimulation with ΔRT and in 50% of the individuals when stimulated *in vitro* with wild type AT-2 inactivated. HIV-1- specific response rates for CD4+ T-cells expressing IFN-γ using the ICS assay were found to be significantly higher after ΔRT stimulation (p = 0.017). By contrast, percentages of specific T cells expressing other cytokines such as IL-2 or TNF-α were similar against both stimuli (Fig. 2B). For CD8+ T-cell responses, response rates observed were 31% and 25% after ΔRT and wild type AT-2 inactivated stimulation, respectively. Lastly, positive responses detected simultaneously in both CD4+ and CD8+ T cell subsets were only observed in two individuals (12.5%). As previously mentioned, a pool of peptides encompassing part of the p24 viral protein was used as a control of HIV-specific T cell responses. As expected, this control induced the highest values for all the cytokine-specific T cells evaluated in both lineages (CD4 and CD8) when compared to virus particles (Fig. 2B).

### Differential Responses against Wild Type AT-2 Inactivated and ΔRT Defined Three Different Patterns of HIV+ Individuals

Analysis of the immune response elicited against wild type AT-2 inactivated and ΔRT viruses showed a surprisingly result: each patient responding to wild type virus also responded against ΔRT virus, but not the other way around (i.e. 0% of patients responded only against wild type AT-2 inactivated). This data allow us to divide the study population into three different groups attending to the profile of their *in vitro* response: full-responders, partial-responders and non-responders.

**Figure 3:**
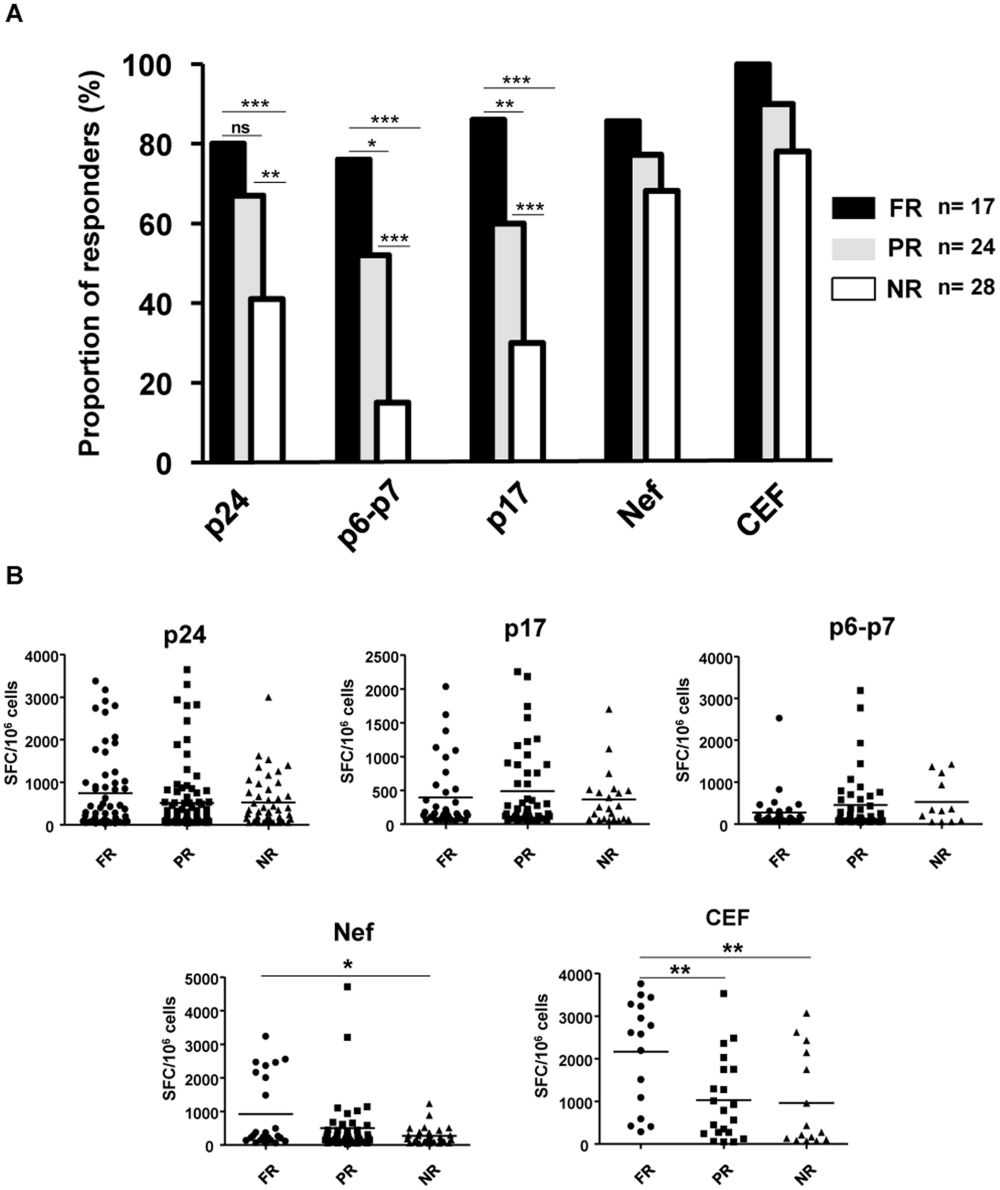
Frequency of IFN-γ-ELISPOT positive responses against different pools of peptides. (**A**) Population analyzed has been divided attending to their positive response against WT+AT2 and/or ΔRT immunogens into three different groups (FR: full-responders; PR: partial-responders and NR: non-responders). The response obtained against overlapping pools of peptides from Gag and Nef showed a different pattern between established groups. Gag response was predominant in responder individuals (see results). CEF peptide pool was used as an HIV-independent peptide stimulus. (**B**) Magnitude (SFC/10^6^ PBMC) of HIV-1-specific T-cell responses against Gag (p24, p17 and p6-p7) protein was not affected among groups whereas Nef present a significant reduction in non-responder individuals. Epitopes from human cytomegalovirus, Epstein-Barr virus and influenza virus (CEF peptide pool) showed a higher magnitude in responder individuals. Statistically significant differences are shown (*p<0.05; **p<0.01; ***p<0.001).

Attending to the mentioned categorization of individuals, the ELISPOT response obtained using overlapping HIV peptide pools encompassing Gag and Nef proteins resulted in a different breadth pattern (Fig. 3A). Full-responders individuals have a higher rate of response against Gag peptides (p24, p17 and small proteins) (76–86%) than partial-responders (52–67%) and non-responders (15–41%), respectively whereas no difference were seen in terms of the magnitude of the Gag specific T cell response (Fig. 3B). On the other hand, Nef and HIV independent peptide pools (CEF Citomegalovirus, Epstein-Barr and Influenza) elicited similar rate of response in the three categories of patients with a significant reduction in the total magnitude (SFC/10^6^cells) of the response mainly in the group of non-responders individuals (Fig. 3B).

**Figure 4:**
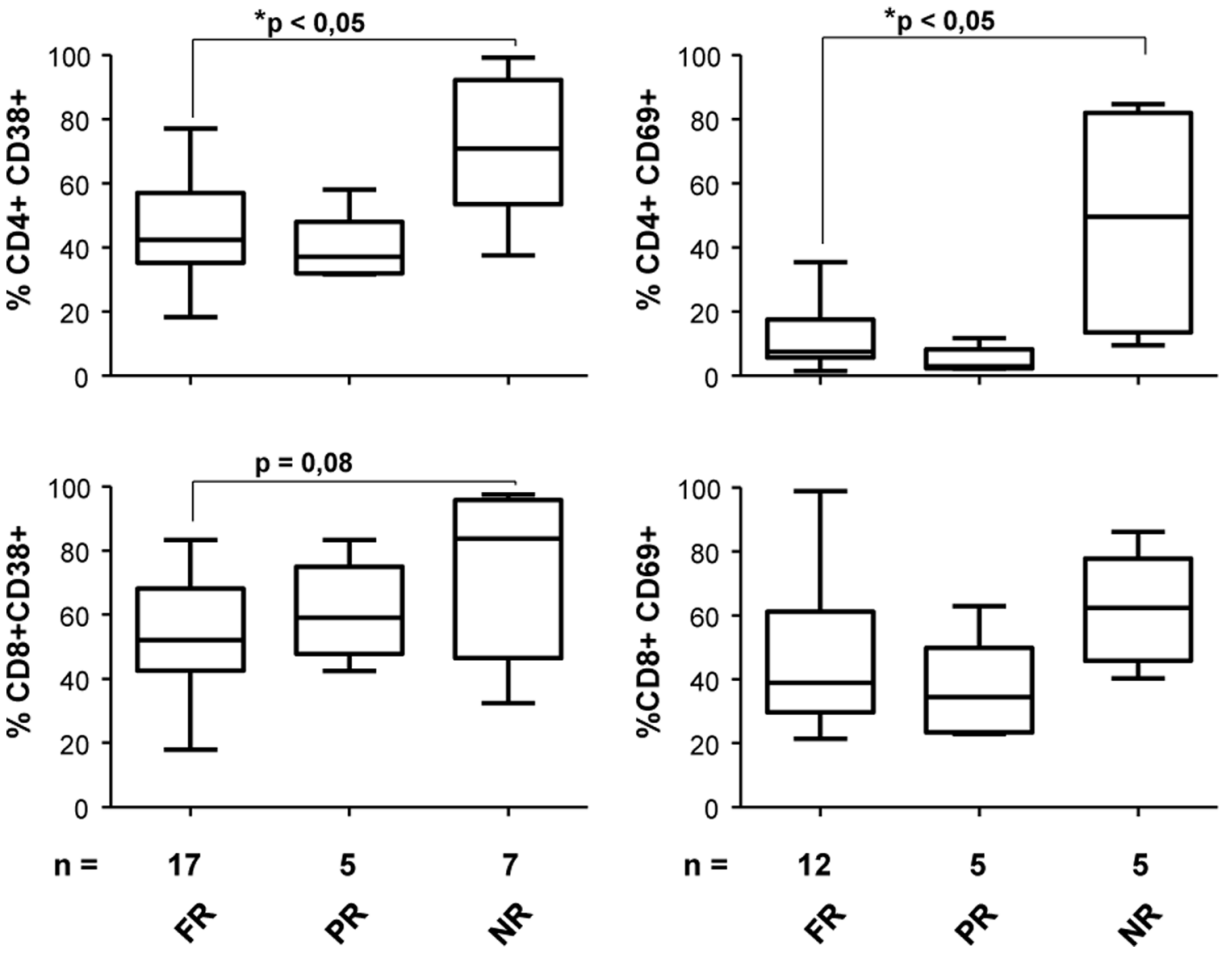
Percentage of expression of activation markers (CD38 and CD69) in CD4 and CD8 T cell subsets. Extracellular staining revealed that baseline activation levels varied significantly between full-responders and non-responders HIV-infected individuals in CD4+CD38+ and CD4+CD69+ subsets. Mann–Whitney P value is shown.

We next assessed activation (CD38, HLA-DR and CD69) and senescence markers (CD28) on T lymphocytes to characterize more accurately immunological features of the three established study groups of HIV-infected patients. Extracellular staining revealed that baseline activation levels varied significantly between full-responders and non-responders individuals in CD4+CD38+ and CD4+CD69+ T cell subsets (p<0.05) whereas were similar when full and partial-responders were compared. Activation markers on CD8+ T cells showed a similar tendency (Fig. 4). Moreover, CD28 and HLA-DR surface markers were similarly expressed in all the individuals tested in both CD4+ and CD8+ T cell subsets (data not shown).

### HLA Protective Alleles, Gender and Viral Load Levels Correlated with the Elicited Response

Next we search for demographic and clinical parameters that could be involved in the different pattern of T cell response observed against viral particles. As shown in Table 1 and after dividing HIV patients in the three different groups depending on the T cell response profile found, we observed that full-responder subjects comprised a significant higher percentage of women (41.2%) than partial-responders (20%) and non-responders (3.7%). Related with this bias and the way in which HIV transmission takes place in women in this cohort (preferentially drug users) [9] risk groups were also slightly different but without statistical significance. Age was not related in any case with the response elicited by the previously defined study groups.

**Table 1:** Demographic characteristics of the studied groups.

	*Full-responder (n = 17)*	*Partial-responder (n = 25)*	*Non-responder (n = 27)*
Sex (p = 0,008**)			
Male	**10 (58.8%)**	**20 (80%)**	**26 (96.3%)**
Female	**7 (41.2%)**	**5 (20%)**	**1 (3.7%)**
**Age in years**			
Median	**43.5**	**37**	**39**
Range	**29–64**	**22–61**	**27–64**
**Risk group**			
Homosexual	**8 (47%)**	**17 (68%)**	**20 (74%)**
Drug users	**4 (23.5%)**	**2 (8%)**	**4 (14.8%)**
Heterosexual	**4 (23.5%)**	**6 (24%)**	**3 (11.2%)**
Hemophilia	**1 (5.9%)**	**0**	**0**

Additionally, different clinical parameters such as plasma viral load, NADIR CD4+, current CD4+ and CD8+ T cell counts, VHC prevalence and HAART (high active antiretroviral treatment) duration were considered (Table 2). We found that plasma viral load was significantly lower in full-responder individuals, surprisingly VHC prevalence did not affected the *in vitro* T cell response. On the other hand, CD4 and CD8 T cell counts, NADIR CD4 T cells, and median time of HAART were similar in all the three groups pre-established of patients.

**Table 2:** Clinical parameters of the studied groups.

		*Full-Responder (n = 17)*	*Partial-responder (n = 25)*	*Non-responder (n = 27)*
Plasma HIV RNA, median (IQR), copies/ml				
		**50 (47–977)**	**1393 (50–4183)**	**508 (50–9222)**
CD4+ T-cell count, mean (95% CI), cells/mm3				
	**767 (587–947)**		**756 (630–883)**	**726 (595–857)**
NADIR CD4+, mean (95% CI), cells/mm3				
	**447 (377–517)**		**482 (390–573)**	**453 (381–526)**
CD8+ T-cell count, mean (95% CI), cells/mm3				
	**1041 (663–1418)**		**1173 (935–1411)**	**1223 (1069–1377)**
Duration of HAART treatment prior to PBMC sample*, median (IQR), months				
	**61 (45–120)**		**56 (30–105)**	**48 (24–76)**
Prevalence of infection by VHC (%)				
	**31**		**9**	**19**

Finally another host factor associated with durable control of HIV or better response to vaccines [10], [11] is the presence of certain HLA class I alleles, particularly HLA-B27and B57 [12]–[16]. Other HLA-B alleles have also been associated with delayed disease progression or lower viral loads, including HLA-B15, B51, and B58 [17], [18]. We then evaluated HLA class I frequencies in 38 patients from our study where HLA typing was available. In our cohort among full-responders, 40% of the subjects expressed either HLA-B27 or B57, while only 23% of non-responders carried these alleles. As expected in a chronic asymptomatic cohort with a good prognosis other protective alleles such as HLA-B15, B51, or B58, were common in both groups (24% in full-responders vs 28% in non-responders; Table 3) In any case the statistical analysis performed using the Chi-square test to determine if this difference between both populations was noteworthy, revealed that it was not significant (p>0.05).

**Table 3:** HLA-B subtypes carrier and allele frequencies in the asymptomatic population analyzed.

	Responder population (n = 17)				Non-responder population (n = 21)		
		n	Carrier frequency	Allele frequency	n	Carrier frequency	Allele frequency
*B27*		4	23%	11%	2	9%	4.5%
*B5701*		3	17%	8.5%	3	14%	7%
*B15*		2	12%	6%	4	18%	9%
*B51*		1	6%	3%	1	5%	2.5%
*B58*		1	6%	3%	1	5%	2.5%

Additionally we found no association between protective HLA-B status and IFN-γ production to any HIV peptide pool or viral particle tested in either full-responders or non-responders individuals (data not shown). Thus, our data showed that there was no apparent relationship between protective HLA-B alleles and the capacity of HIV-specific CD4+ or CD8+ T-cells to express IFN-γ after stimulation with the viruses tested.

Lastly, and as our division of HIV patients according to the T cell response profile observed also seems to be quite similar to the standardized definition of the various clinical progression profiles: elite controllers (EC), viremic controllers (VC) and chronic progressors with or w/o treatment (CP), we looked at the composition or proportion of EC, VC and CP in our three groups of study. In this comparison EC raised up to 29.4% in full-responders individuals, whereas in partial-responders and non-responders this percentage decreased to 13% and 0%, respectively. On the other hand CP were more over-represented in the group of non-responders (63%) than in those who respond at least to one construction (29–35%) (Figure S1).

## Discussion

The development of a vaccine to control the HIV/AIDS pandemic has become an urgent priority and an international goal. Big efforts have been done toward the development of vaccines that induce effective T cell responses apart from produce antibodies that can broadly neutralize HIV-1 [19]. In this context validated assays and reagents to detect vaccine-induced HIV-specific T cell responses has emerged as a powerful tool to identify either potential future immunogens and epitope specificities of multiple cytokine-secreting CD4+ and CD8+ T cells from cryopreserved PBMC [20]. Although, there is no agreement on the best methods or reagents for assaying the breadth and magnitude of HIV-specific T cell responses. Many studies have used IFN-*γ*-ELISPOT assays and/or Intracellular Cytokyne Staining (ICS) together with overlapping peptide sets to screen T cell responses [21], [22]. Many factors need to be considered before starting this approach as peptide length, purity, overlap, or pool size. All these aspects remains actually controversial because of the obtained differences in the elicited response [23], [24]. It is well known that 20-mer overlapping peptides detect CD4 T cell responses in preference to CD8 T cell responses, whereas shorter peptides of 15-mer seem to be more efficient for stimulating both lineages (CD4 and CD8) [22]. If epitopes have been previously mapped, optimal HLA class I-restricted, 9-mer peptides can be used to increase sensitivity and specificity of the CD8 T cell responses [6]. In any case and independently of the selected approach, ELISPOT and/or ICS assays require large numbers of PBMCs and expensive libraries of peptides. These libraries need to pass previously a quality control to ensure they do not induce unspecific results [7]. In addition, these peptide-based approaches have other drawbacks, the extent to which the sequence of a reference strain as well as the length and degree of peptide overlap influence the detection of T cell responses is not well characterized [23], [25].

In our study we have evaluated a defective recombinant vector (892 bp of the RT gene are deleted; pNL4-3/ΔRT) based on the HIV-1 genome to be used as a reagent for detecting HIV specific T cell responses and as future candidate to serve as immunogen in therapeutic vaccine trials. Since HIV is a highly variable retrovirus and differences in sequence between two viruses within a subtype can be as high as 30%, constructing consensus sequences has been proposed as an alternative to cover the required diversity of subtypes [26], [27]. Moreover these vectors have great potential because they could carry optimized sequences of different HIV-1 genes that could improve the obtained response.

Antigen processing and presentation by DC is required for optimal induction of HIV-1-specific T cell responses, which are essential in controlling infection *in vivo*. This process is a complex mechanism that involves many elements of the antigen processing machinery localized in the endosomal compartment and/or the cytosol [28]. Several works have showed that, for DC, little amounts of antigens are sufficient for presentation on MHC molecules and activation of T cells. Some of these approaches have been done using antigens from non-replicating viruses, including aldrithiol-2 (AT-2)-inactivated HIV-1 [29], [30]. During HIV-1 infection, most of circulating virus is not infectious [31]. Taking advantage of this phenomenon, defective and non-replicating virions may constitute an interesting source of HIV-1 antigens for activation of HIV-1-specific CD4+ and CD8+ T cell responses. In fact, our defective virus is an infectious but non-replicating virus which conserves conformational and functionally intact surface envelope proteins that can interact with T cells as well as with surface receptors present on circulating dendritic cells and monocytes. Therefore, we hypothesized that PBMCs may also be useful to present antigens from fusion competent nonreplicating virus (i.e. wild type AT-2 inactived and ΔRT) [30], [32] and then to detect HIV-specific T cell responses. The designed assay resulted faster and cheaper although in comparison with DC the percentage of positive responses was relatively lower [33]. Nevertheless our defective construct showed a better response (in terms of frequency and magnitude; see fig. 1) in comparison with AT-2 inactivated wild type virions. The potential mechanisms responsible of such differences have been discussed elsewhere [8] but it seems that probably, the maturation status of our defective particle could imply a major stability which allows increased peptide presentation and processing by APC. This result is really interesting because the magnitude of a vaccine-induced responses may be related to the potency and frequency of immunization, which may also influence durability of the response [34]. As Pantaleo et al. [35] describe the broader the antigen pool the wider the T cell repertoire that can be mobilized. All this data confirm that ΔRT can serve as an efficient source of antigen for priming both CD4+ and CD8+ T cells (fig. 2A) and therefore potentially function as a relevant source of antigen for priming *in vivo* [28]. Another important advantage derives from the fact that AT-2 treatment required previous studies of non-toxicity in animal-models and GMP **(**Good manufacturing practice) conditions that make it not suitable for be used in humans in our country (personal communication from the AEMPS, Agencia Española del Medicamento y Productos Sanitarios). ΔRT could be included in clinical trials without attending all these requirements. Moreover, and as we have shown, ΔRT could be considered as an extremely useful reagent to detect the presence *in vitro* of HIV specific T cell responses. Some of the advantages that we observed using these virions were related with the feasibility, simplicity and effective cost of the production of both the defective virus and the assay. In fact, the use of our defective viruses would considerably reduce the amount of cells required to perform an initial screening of T cell responses. Moreover, and although stimulation with defective virions resulted in lower levels of IFN-gamma T cell responses in HIV-1-infected individuals, the response rate was similar to the obtained after stimulating with HIV peptides.

Diverged response obtained between techniques (ICS and ELISPOT), although previously described [36], could be explained by many reasons like different time of incubation between techniques (see Methods, 6 hours instead of 16 hours), different frequencies of specific-T cells response or different technical sensitivity threshold. Concerning the different time of stimulation, and although we can not be certain than longer incubation periods could increase the response to the virions tested, it has been described that measurement of IFN-g production is often optimal after 6 h of simulation and not significantly improved with increased incubation times [37]. So, we considered that a standard 6h-ICS could be sufficient to allow the detection of HIV-specific T cells. On the other hand, it is well known that frequencies of T cells recognizing individual antigens are very low. As a result of the clonal expansion associated with infections, the frequency of antigen-specific effector cells can rise up to 1∶100, but these frequencies normally settle in the range of 1∶10,000 for effector memory or memory [38], [39]. It is well established that ICS measures intracellular cytokine whereas ELISPOT detects the actually released product. Only the last one is biologically functional, because cytokine production can be post-translationally modified, meaning cytokine that is synthesized by a cell but not released, will not exert any effector function *in vivo*. This premise confirmed that our screening mainly conducted by the ELISPOT assay is a good indicator of immune T cell response. Nevertheless, we should consider that using a PBMC ELISPOT results include all IFN-g-producing cells within the PBMC sample and not only CD3+ T cells as by ICS.

Differential response against NL4-3+AT-2 and NL4-3/ΔRT allowed us to define three different groups of HIV-infected patients (full-responders, partial-responders and non-responders) in a cohort of chronic asymptomatic HIV positive individuals. This quite homogeneous population was divided in these three categories attending to the elicited response and moreover, this division seems to adequately correlate with different aspects of the immunological state and disease progression. For instance the higher rate of response against Gag peptides (76–86%; Fig. 3A) observed in full-responders as well as the highest capacity to respond to HIV independent peptides (CEF: CMV, Epstein-Barr and Influenza) (Fig. 3B), and lastly the presence of lower levels of T cell activation, which constituted a key aspect of HIV pathogenesis [40], and lower levels of HIV viral replication, suggest that HIV-infected individuals able to respond to our defective viral particle should have a preserved functional immune system and consequently a better prognosis and a more delayed clinical progression. In fact, there was a greater percentage of HIV elite controllers among the full-responders to our viral particles, additionally, and as it has been described, a combination of host genetic and viral factors supports current clinical definitions that discriminate among patterns of HIV-1 progression [41]. In our case, 40% of the full-responder individuals expressed HLA-B27 or B57, HLA class I alleles associated with durable control of HIV [15], [16], [42] and better response to vaccines [10], [11], whereas only 23% of non-responders subjects carried these alleles. Interestingly, we found a significant higher rate of women in responder population (41,2%), which were HLA-B27 carriers in a high frequency (43%). This result could be responsible of the differential response elicited that could be attributable to the presence or absence of this protective allele [34]. So, the correlations observed between the elicited response and the immunological state of the patients could be useful to validate in one-step assay the eligibility of future candidates to be included in therapeutic or prophylactic vaccine trials.

In summary this kind of procedures and reagents allowed *in vitro* immunogenicity comparisons between potential future immunogens or vaccine candidates. IFN-γ ELISPOT and intracellular cytokine staining assays have been applied in the evaluation of T cell responses against defective and/or inactivated non-replicative viral particles (ΔRT or wild type AT-2 inactivated respectively). Although we have to be cautious because a similar approach during the evaluation of the Step trial detected high response rates (90% in vaccinees after three immunizations) and the persistence of the CD8+ T cell responses after 1 year, but it did not predict vaccine efficacy [43]. The possibility to increase the immunogenicity in a safer and more efficient way with these retrotranscriptase defective virions that could carry optimized sequences of different HIV-1 genes and offer the possibility of construct chimeric viral particles including sequences of autologous HIV virus, postulated them as a useful tool. They could be used as an effective immunogen or as an additional or alternative reagent for screening HIV-1-specific T cell responses in both HIV seropositives and vaccinees. These viral particles were useful to screen in a very simple manner our cohort and demonstrated a good correlation with clinical and immunological features. Finally, the implications of using reagents needing to be internalized and processed by a cell, versus peptides for which no processing is required, and peptides concentrations that are usually used in excess, also need to be considered.

## Materials and Methods

### Generation of Virus Stocks

The X4 HIV strains used were the NL4-3 and the NL4-3/ΔRT [8]. R5 HIV strains were the NL4-3/ΔRT/Δenv[BaL] and NL4-3/ΔRT/Δenv[AC10], obtained by modifications of the NL4-3/ΔRT (patent code EP 11382103.7).

Virus stocks were produced by transfecting 293-T cells with different vectors derivated from the clone HIV-1 NL4-3 using the calcium-phosphate method method (ProFection® Mammalian Transfection System; Promega, Madison, WI) according to the manufacturer’s instructions. 5 µg of each purified DNA constructs (Qiagen, Valencia, CA) were used for the transfection. The supernatants were harvested two days and clarified by centrifugation at 500 g/4°C for 10 min. Virus stocks were inactivated with AT-2 treatment when required. To this aim a 100 mM stock solution in DMSO (SIGMA-ALDRICH) was prepared and added directly to virus to produce a 1 mM AT-2 solution and treated at 4°C over night [44], [45]. Viral supernatants were concentrated through ultracentrifugation (Sorvall Ultra Series WX Ultra 80; Thermo Fisher Scientific, Asheville, NC). Viruses were quantified by determining the concentration of p24 in the supernatant by an antigen (Ag) capture assay (ELISA; Innogenetics NV, Gent, Belgium).

### Clinical Specimens

All the experiments made for measuring ELISPOT response and Intracellular Cytokine staining (ICS) have been done using cryopreserved PBMC from up to 69 asymptomatic HIV positive individuals in a cross-sectional study from treated and untreated chronic asymptomatic HIV-1 individuals with baseline CD4^+^ T lymphocyte counts >500 cells/mm^3^ and plasma viral loads ranging from 50–10,000 HIV-1 RNA copies/mL (Hospital Clinic, Barcelona, Spain). PBMCs were isolated by centrifugation through a ficoll-hypaque gradient and cryopreserved. All cell cultures were performed at 37°C in a fully humidified atmosphere with 5 % CO_2_ in air. All the individuals gave informed written consent and this study was reviewed and approved by the Institutional Ethical Committee board of Hospital Clinic (Barcelona, Spain).

### Enzyme-linked Immunospot (ELISPOT) Assay for IFN-γ Release

Ex vivo measurement of T cells for IFN-γ production was undertaken by the ELISPOT assay as previously described [46], [47]. Briefly, ninety-six-well plates (Multiscreen Millipore, Bedford, MA) were coated overnight at 4°C with 15 µg/ml of anti-IFN-γ mAb 1-D1K (Mabtech, Stockholm, Sweden) in coating buffer (Na2 CO3 0.1 M, pH 9.6). The plates were washed four times and blocked with RPMI 1640/10% FBS. PBMCs were added to the coated plates during 16–18 h at a final concentration of 1×10^5^/100 µl. We have used peptide pools obtained from NIH AIDS Reagent Program, spanning the entire HIV-1 consensus B Gag and Nef proteins sequences. They consisted of 15-mer peptides overlapping by 11, grouped in pools of 10–12 each (5 of gag p24, 3 of gag p17, 2 of gag small proteins, and 2 of nef). All the pools were used at a final concentration of 2 µg/ml. As described above different constructions have been also used to evaluated this issue (pNL4-3+AT-2; pNL4-3/ΔRT; pNL4-3/ΔRT+AT-2; pNL4-3/ΔRT/ΔEnv[AC10] and pNL4-3/ΔRT/ΔEnv[Bal]). In all cases we evaluated a dose of 200 ng/ml of HIV-1 p24. Additionally a negative control (RPMI/10%FCS) and two positive controls such as a pool of MHC class I-restricted T cell epitopes from human cytomegalovirus, Epstein-Barr virus and influenza virus (CEF, Mabtech) and phytohemagglutinin (PHA 2 µg/ml; Sigma) were performed. The cells were lysed with PBS/0.05% Tween 20, and the wells were incubated for 3 h with 1 µg/ml of biotin-labelled, anti-IFN-γ mAb 7-B6-1 (Mabtech). To visualize the spot forming foci (IFN-γ-secreting cells), the wells were washed six times and treated during 1 h with streptavidin-alkaline phosphatase (Mabtech). After this incubation the wells were washed again and 100 µl/well of chromogenic alkaline phosphatase conjugated substrate (BioRad, Hercules, CA, USA) were added. The spots were clearly visible in less than 30 minutes ***avoiding*** direct ***light*** exposure. Spot forming cells (SFC) were counted using an AID ELISPOT reader (Autoimmun Diagnostica GmHb, Germany). Results were considered positive if the number of SFC/10^6^ PBMC in stimulated wells was twofold higher than that in unstimulated control wells, and if there were at least 50 SFC/10^6^ PBMC after background subtraction.

### Activation Markers and Surface Staining

Subpopulations of CD3+, CD4+, and CD8+ cells were determined by four-color flow cytometry. The following monoclonal antibodies were used: CD3-peridinin chlorophyll protein (PerCP), CD4- allophycocyanin (APC), CD8-APC, CD28-fluoroisothiocyanate (FITC), HLA DR-FITC, CD57-FITC, CD69-phycoerithrin (PE) and CD38-PE (all from Becton Dickinson, Mountain View, CA). Mouse immunoglobulin isotypes conjugated with Per-CP, PE, FITC or APC were always used as negative controls for nonspecific binding. The stained cells were analyzed on a FACSCalibur (Becton Dickinson, San Jose, CA) flow cytometer. Lymphocytes were gated on the basis of forward and side scatter parameters. The gating region was referred to an FL3/SS histogram, where an FL3+ (CD3+) region was defined. This region was further analyzed for the expression of FL1, FL2 and FL4. Data were analyzed using FlowJo Software (Tree Star).

### ICS Assay

Intracellular staining profile were analyzed by ICS as described previously elsewhere [48]. Briefly, cryopreserved PBMC were thawed, washed and resuspended in RPMI1640 with v/v 10% FCS and then incubated overnight. Cells were seeded on M96 plates and stimulated with NL4-3 and NL4-3/ΔRT virions at a final concentration of 200 ng/ml. A p24 peptide-pool and PMA-Ionophore stimulus were also used as positive controls (2 µgr/ml and 50 ng/ml-500 ng/ml at a final concentration, respectively). Protein transport inhibitors Monensin (GolgiStop, BD Pharmingen) were added after set-up for the different stimuli and cells were incubated at 37°C, 5% CO2 during 6 hours. After stimulation, cells were washed, stained for the surface markers, fixed, permeabilized using the BD Cytofix/CytopermTM Kit (Becton Dickinson) and stained intracellularly using the appropriate fluorochromes. Cells were acquired using a FACsCanto flow cytometer (Becton Dickinson) and the number of events ranged between 2×10^5^ and 10^6^.

Intracellular staining have been analyzed as follows: lymphocytes were gated on a forward scatter area versus side scatter area pseudo-color dot plot, then on CD3+ (APC-Cy7), and from there, CD4+ (PE-Cy7) and CD8+ (PerCP Cy5.5) subsets are identified. Each of those two subsets producing IFN-γ, TNF-α and IL-2 were subsequently identified in the corresponding channel (APC, PE and FITC respectively). Percentages of stimulated cytokine-producing T cells were considered positive when values were three times higher than the non-stimulated background.

### HLA Typing

HLA class I typing was carried out at Servicio de Inmunología, CDB (Hospital Clinic i Provincial de Barcelona, Spain). Briefly, HLA loci were molecularly typed with DNA extracted from PBMCs using QIAmp Blood Kit (Qiagen, Valencia, CA). The HLA class I genotype was determined by reverse sequence-specific oligonucleotide (SSO) (RELITM Dynal, Madrid, Spain). Allele definition was automatically assigned by the RELITM SSO Pattern matching program software and was manually supervised [46].

### Statistical Analysis

Median and interquartile range (IQR) were used to describe each of the continuous variables analyzed. Data analysis and comparisons for the different parameters were made using parametric (t-student) and nonparametric (e.g. Mann-Whitney or Wilcoxon) tests as appropriate. For comparisons of multiple groups the Kruskal-Wallis test was performed with correction using Dunn’s multiple comparison test. Statistical analysis was performed using SPSS 18.0 (Windows) software (SPSS Inc, Chicago, IL) and GraphPad Software, San Diego California USA (GraphPad Prism version 5.00). For all analyses, the level of significance was set at p<0.05.

## Supporting Information

Figure S1 **Distribution of the different clinical progression profiles (EC, VC and CP+HAART) within the groups described previously (FR: full-responders; PR: partial-responders and NR: non-responders).**
(TIF)
