# Supplementary figures and images for: Use of RT-Defective HIV Virions: New Tool to Evaluate Specific Response in Chronic Asymptomatic HIV-Infected Individuals
Source: PLoS One. 2013 Mar 14;8(3):e58927. doi: 10.1371/journal.pone.0058927 (PMC3597525; doi:10.1371/journal.pone.0058927)

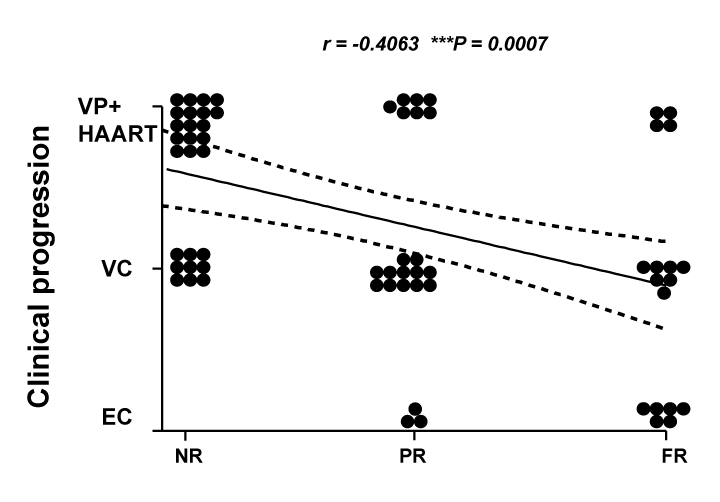

Supplement: Figure S1 — Distribution of the different clinical progression profiles (EC, VC and CP+HAART) within the groups described previously (FR: full-responders; PR: partial-responders and NR: non-responders). (TIF) [file pone.0058927.s001.tif]
